# Exploring Decision-Making Strategies in the IOWA Gambling Task and Rat Gambling Task

**DOI:** 10.1192/j.eurpsy.2023.296

**Published:** 2023-07-19

**Authors:** C. Hultman, N. Tjernström, S. Vadlin, M. Rehn, K. W. Nilsson, E. Roman, C. Åslund

**Affiliations:** 1 Department of Public Health and Caring Sciences, Faculty of Medicine, Uppsala University, Centre for Clinical Research, Västmanland Hospital, Region Västmanland, Uppsala University, Västerås; 2 Department of Pharmaceutical Biosciences, Uppsala University, Uppsala; 3 Centre for Clinical Research, Västmanland Hospital, Region Västmanland, Uppsala University, Västerås, Sweden; 4 School of Health, Care and Social Welfare, Mälardalen University, Centre for Clinical Research, Västmanland Hospital, Region Västmanland, Uppsala University, Västerås; 5 Department of Anatomy, Physiology and Biochemistry, Swedish University of Agricultural Sciences, Department of Pharmaceutical Biosciences, Uppsala University, Uppsala, Sweden

## Abstract

**Introduction:**

Impairments in decision-making processes are believed to play an important role in both substance use disorders and behavioral addictions. Clinical and pre-clinical experimental testing provide complimentary insights on the psychobiological mechanisms of decision-making. The IOWA Gambling Task (IGT) assesses decision-making under ambiguity and risk, in which individuals are faced with four card choices associated with varying monetary reinforcer/loss contingencies. The rat Gambling Task is a pre-clinical version using palatable reinforcers as wins and timeouts mimicking losses. However, studies with interspecies comparisons in these tasks are lacking, but important to facilitate translation of information that may help unravel the complex processes of decision-making and generate clinical advances.

**Objectives:**

This study explores decision-making strategies among humans and rats performing the IGT and rGT.

**Methods:**

A total of 270 young human adults performed a computerized version of the IGT, and 72 adult outbread male Lister Hooded rats performed the rGT. Performance was assessed and explored by normative scoring approaches and subgroup formations based on individual choices.

**Results:**

Results showed that most humans and rats learned to favor the advantageous choices, but the overall level of performance differed considerably. Humans displayed both exploration and learning as the task progressed, while rats showed relatively consistent pronounced preferences for the advantageous choices throughout the task. Nevertheless, variability in individual choice preferences during end performance were evident in both species.

**Conclusions:**

Results are discussed in relation to procedural differences impacting performance and potential to study different aspects of decision-making. This is a first attempt to provide formal evaluation of similarities and differences regarding decision-making processes in the IGT and rGT from an explorative perspective.

**Disclosure of Interest:**

None Declared

